# Have New Plate Designs Reduced the Rate of Hardware Removal Following Midshaft Clavicle Fracture Fixation?

**DOI:** 10.3390/jcm14186351

**Published:** 2025-09-09

**Authors:** Maria Oulianski, Yoram Weil, Omer Ben Yehuda, Rami Mosheiff, Mahmoud Jammal

**Affiliations:** Orthopedic Department, Hadassah Hebrew University Hospital, Jerusalem POB 12000, Israel; oulianskim@gmail.com (M.O.); weily@hadassah.org.il (Y.W.); omer.benyehuda@gmail.com (O.B.Y.); ramim@hadassah.org.il (R.M.)

**Keywords:** clavicle fractures, pre-contoured plates, hardware removal, fixation

## Abstract

**Objectives**: Operative fixation of displaced midshaft clavicle fractures has become increasingly the more acceptable choice of care in recent years, based on evidence supporting its effectiveness. However, this practice presents challenges due to the complex S-shaped morphology of the clavicle and its subcutaneous location. Despite the introduction of anatomically pre-contoured plates, achieving optimal implant-to-bone fit remains difficult, prompting the development of newer plate generations. The aim of this study was to compare the hardware removal rates of second-generation 2.7 mm thinner plates (SGPs) with those of first-generation 3.5 mm plates (FGPs). **Methods**: A retrospective comparative cohort study was conducted at a level one trauma center. A total of 187 patients received FGPs, and 67 received SGPs, both positioned on the superior bone surface. All surgeries were performed by fellowship-trained surgeons, and patients were followed for at least one year. Data were extracted from medical records and the PACS system. **Results**: The patients’ demographics (age: 32.86 vs. 33.14 years; gender: 16.85% vs. 14.92% female) and fracture type (AO/OTA) were similar between the two groups. The rate of implant removal (20.1% vs. 20.9%) did not differ significantly between groups. Complications included nonunion (1.6% vs. 1.7%, statistically not significant) and infection (three cases in the FGP group, none in the SGP group). **Conclusions**: Despite the high success rate of clavicle fixation procedures, the superior clavicular plate removal rate remains significant, regardless of the implant design.

## 1. Introduction

Clavicle fractures are frequent injuries, representing 2.6–10% of fractures in adults, of which midshaft fractures are the most common, accounting for approximately 81% of all clavicle fractures. Middle-third clavicle fractures have long been managed conservatively with immobilization. Some patients, especially those with completely displaced or shortened clavicle fractures, are now thought to have an increased risk of nonunion or symptomatic malunion [1,2]. Thus, the rate of operative fixation for midshaft clavicle fractures has increased dramatically over the past decades. Recent evidence, including findings from multiple randomized controlled trials, demonstrated a reduction in nonunion and symptomatic malunion rates following plate fixation of clavicle shaft fractures compared to non-operative treatment. As a result, a steep rise in the surgical management of clavicle shaft fractures has been observed in developed countries in recent years [1,2,3].

**Figure 1:**
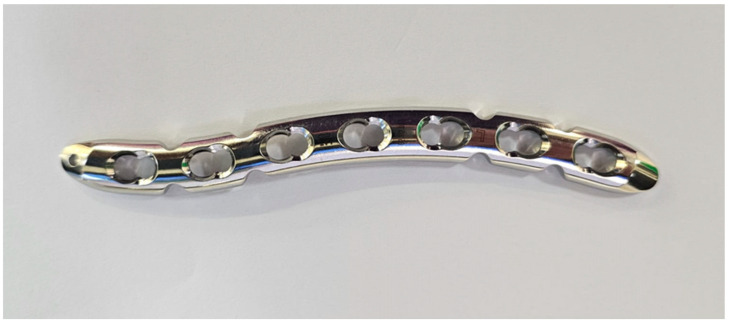
Left side of 3.5 mm LCP plate.

However, this practice is not devoid of challenges and complications. The clavicle displays definite gender- and side-specific anatomical features in terms of length, diameter, curvature, and calcium concentration; its unique S-shaped bony anatomy exhibits significant variance across gender, ethnicity, patient stature, and age [4]. Additionally, as the clavicle is a subcutaneous bone, the prominence of implanted plates can lead to irritation and discomfort. With the increasing preference for operative plate fixation, the first generation of pre-contoured anatomical plates was developed, typically accommodating a 3.5 mm screw size (Figure 1). Despite their biomechanical superiority over generic plates, their initial designs failed to account for the wide variability of the clavicle bone anatomy. Several studies have reported suboptimal fit, particularly with superior plate designs [5,6,7]. The apex of the superior bow of the clavicle is typically located along the lateral aspect of the bone, whereas the medial aspect of the superior surface of the clavicle remains relatively flat, making it an ideal plating surface. Thus, pre-contoured anatomical plate fits in the medial three-fifths of the clavicle but does not fit as well laterally [5,6].

Other solutions have been proposed to address these problems, including anteroinferior plate positioning, multiple mini-fragment plates, and recently improved plate designs. Recent innovations have introduced lower-profile plates, smaller diameter screws, and a greater variety of plate shapes tailored to patient height, all aimed at minimizing implant-related complications [8,9].

Plate construct prominence is influenced by two variables: plate thickness and how well the plate fits the bow and curvature of the clavicle. New plate shapes are designed to accommodate the bow and curvature of the clavicle at corresponding fracture locations to achieve an enhanced plate-to-bone fit [8,9,10].

Despite these potential solutions, hardware removal remains common following clavicular plate fixation, with reported rates ranging from 5% to 27%. The primary reasons for implant removal include pain, discomfort, and hardware prominence [10,11,12].

**Figure 2:**
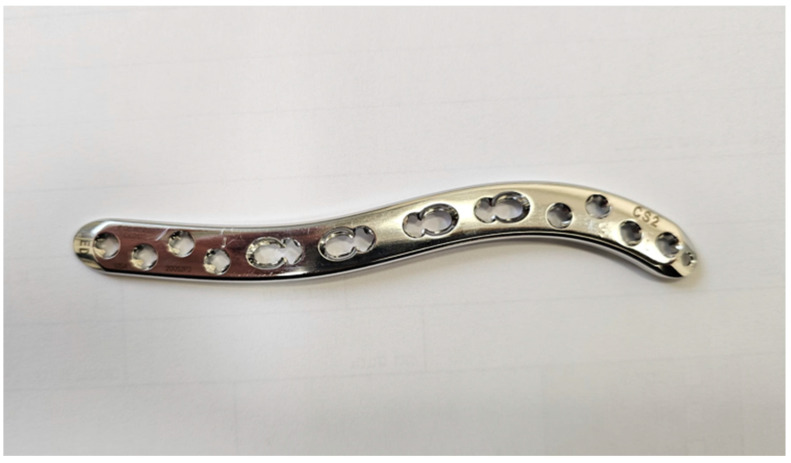
A 2.7 mm VA-LCP plate-CS2.

Over the past three years, the authors have adopted the use of second-generation plates—VA-LCP 2.7 mm clavicle system [13] (Figure 2). These plates, according to the manufacturer, were designed to be thinner by 13–21% than FGPs, with tapered edges and low-profile design, shaped to match the bow and contour of the clavicle for low prominence and enhanced plate-to-bone fit [8].

This study evaluated the hardware removal rate as the primary outcome measurement following the transition to the more anatomically fit plates. The study hypothesized that this transition, incorporating a more anatomical design with increased variability, would result in a lower implant removal rate. The novelty of this study lies in its relatively large cohort size compared to previous reports, combined with a detailed breakdown of the indications for implant removal which stratifies and compares the specific reasons for hardware removal between first and second generations of anatomic superior clavicular plates.

## 2. Patients and Methods

This retrospective, IRB-approved comparative cohort study was performed at a university-based level one trauma center. Between 2017 and 2024, a total of 369 operative midshaft clavicle fracture fixations were performed using superiorly positioned, anatomically pre-contoured plates. All procedures were conducted by fellowship-trained trauma or shoulder surgeons. Inclusion criteria included patients aged 18 years and older, fractures located at the midshaft of the clavicle, AO/OTA “B type”, B1–B3 fractures as the only included fractures, and fixation using a single plate. Clavicular fractures treated with dual plating or intramedullary nails were excluded. Patients with pathological fractures were excluded. All patients had to have a minimum of one year clinical and radiological follow-up postoperatively to be considered for inclusion. In this retrospective study, patients were divided into two groups: the first group includes patients treated with first-generation plates, and the second group includes patients treated with second-generation plates; these plates are both anatomic pre-contoured stainless steel and have properties of reconstruction and low contact plating systems. Of the 369 cases, 251 patients met the inclusion criteria. Among these, 184 patients were treated with 3.5 mm first-generation locking plates (group 1, FGP), while 67 patients received second-generation 2.7 mm plates (group 2, SGP). Then, we checked in every group the number of patients who retained their plates and the number of those whose plates have been removed. In both groups, we assessed age, gender, side of operation, the reason for plate removal if indicated, and complications.

### 2.1. Surgical Technique

The surgical technique was performed according to widely established recommendations as described in the literature. All patients were positioned in the beach chair position for surgery. A superior direct incision was made, and exposure was achieved using sharp dissection. Great caution was taken to prevent clavicular stripping and to save bone vascularity. Provisional reduction was performed using bone clamps or Kirschner wires (K-wires). All clavicles were stabilized via a compression or bridging technique using suitable locking and non-locking screws through the superior plate. In cases of comminuted fractures, butterfly fragments were either fixed or left untreated at the surgeon’s discretion, using 2.7 mm independent lag screws. The platysma muscle was sutured over the plate along with the pectoralis major for appropriate soft tissue closure. The muscle layers were meticulously closed with interrupted sutures to maximize plate coverage, and the skin was approximated using absorbable subcuticular sutures.

Postoperative care consisted of allowing an unrestricted range of motion immediately after surgery, except for avoiding lifting objects heavier than 5 kg. A gradual return to sports was permitted between 6 and 8 weeks postoperatively. Routine follow-up visits were scheduled at 2 weeks, 6 weeks, 12 weeks, 6 months, and 1 year. Standard two-view clavicular radiographs were taken serially at the time of follow-up until fracture healing and bone union. Bony healing was defined as painless range of motion with bridging of at least 3 out 4 cortices seen on three radiographic projections (AP, 30-degree caudal, and cranial views). Data for the study were extracted from electronic medical records (EMRs), and radiographic images were obtained from the Picture Archiving and Communication System (PACS, Synapse FUJIFILM Healthcare Americas Corporation, Lexington, MA, USA).

### 2.2. Statistical Methods

The normality of the data distribution was assessed using the Kolmogorov–Smirnov test. Continuous variables were analyzed using a two-tailed Student’s *t*-test or analysis of variance (ANOVA), as appropriate. Categorical variables were compared using Pearson’s chi-square test. A *p*-value < 0.05 was considered statistically significant. Data was analyzed using IBM SPSS Statistics for Windows (Version 26.0, Armonk, NY, USA).

## 3. Results

The two study groups were comparable in age, gender, operative side, and the use of lag screw fixation (Table 1). Our patient population study included 41 females, 31/184 in the FGP group and 10/67 in the SGP group, i.e., 16.85% and 14.92%, respectively. Most of the patients in both groups were male, about 85%. In total, 127 patients had left-side injuries, and 124 suffered right-side injuries, with 95/184 in the FGP group and 32/67 in the SGP group. The average age of patients was 32 years in both groups. All fractures, besides four (see below), healed both clinically and radiographically without the need for further intervention.

The overall plate removal rate was approximately 20% in both groups, 37/184 in the FGP group and 14/67 in the SGP group, with no statistically significant difference between them (OR: 0.96) (Table 2). Patient-reported reasons for plate removal included pain, prominence, irritation, and/or limited range of motion. The mean time to implant removal was 11 months following the index procedure, with no significant difference between the groups (Table 2). Among the patients who underwent implant removal, 10 were female and 41 were male; however, gender was not a statistically significant predictor for implant removal. Implant removal was significantly more common on the left side in both groups (Table 2). The average age of patients who went through plate removal was 30 years in the FGP group and 28 years in the SGP group, with no significant difference between them. Gender distribution in the removal groups was found to be 8 females out of 37 patients of the FGP group who needed plate removal, compared to 2 females out of 14 patients of the SGP group, 20% and 14%, respectively, without statistical significance. The lag screw used equally in both groups had no impact on plate removal.

Complications after fracture stabilization and before plate removal procedure included four cases of aseptic nonunion, one in the SGP group (1.7%) and three in the FGP group (1.6%), with no statistically significant difference. In group 1, FGP, one patient developed a surgical site infection, which was successfully treated with intravenous antibiotics. Another case in this group involved an infected nonunion that required conversion to a long 2.7 mm plate combined with intravenous antibiotic therapy; this case ultimately healed.

Three more patients treated for deep infections following plate removal after fracture healing needed subsequent antibiotic treatment. No more complications were observed after plate removal surgery in either group.

We conducted a multivariable logistic regression analysis for plate removal, which shows that younger patients (mean 29.4 years) are more likely to have plate removal compared to older patients (mean 33.5 years). Each additional year of age decreases the removal odds by 3% (OR: 0.97 per year). Another finding of the analysis is the effect of lag screws. Lag screw use may reduce removal risk by 48%, with removal rates of 14.5% for fractures fixed by lag screws versus 24.6% for fractures fixed without lag screws (OR: 0.52). No significant differences were observed in removal rates between B1, B2, and B3 fracture types; no other significant findings were found in the multivariable regression.

Analysis of reasons for hardware removal shows different patterns between the groups (Table 3). The most common reported indications for plate removal were irritation (32.7%), pain (22.4%), and hardware prominence (16.3%).

In group 1, the most common reason for removal was irritation (38.9%) followed by pain (25.0%). In group 2, neither irritation nor pain was the leading cause for plate removal; rather, prominence was the leading cause for plate removal (38.5%).

**Figure 3:**
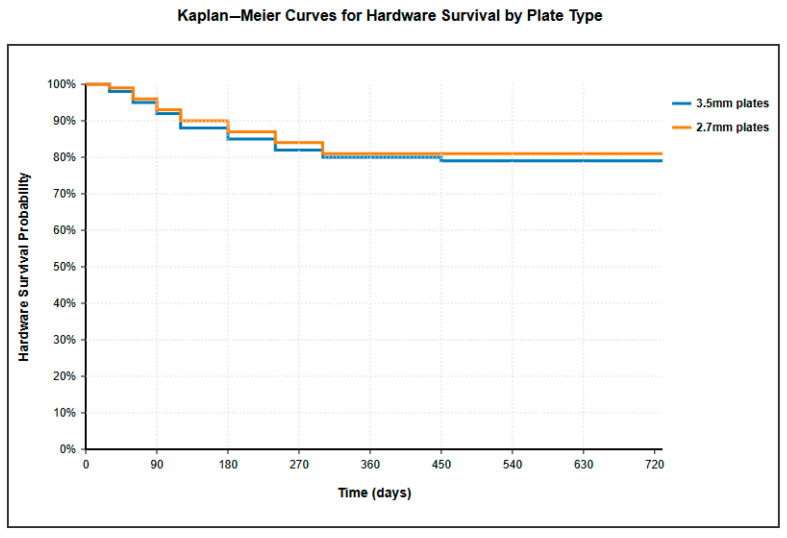
Kaplan–Meier survival curves showing hardware survival probability over time. Blue line = 3.5 mm plates; orange line = 2.7 mm plates. No significant difference between groups (*p* > 0.05).

Kaplan–Meier survival analysis revealed no significant difference in hardware survival between 3.5 mm and 2.7 mm plates (Figure 3). The 2-year hardware survival rates were 79% for 3.5 mm plates and 81% for 2.7 mm plates (log-rank test, *p* > 0.05). Most hardware removals occurred within the first 6 months post-surgery, with both curves reaching a plateau thereafter.

## 4. Discussion

This study did not demonstrate a clear advantage of second-generation anatomical plates over older, bulkier first-generation plates in terms of reducing plate removal rates when it is positioned over the superior clavicular surface. Additionally, union and complication rates after the first fracture stabilization procedure were found to be similar between the two study groups. Another finding of this study was the relatively high rate of implant removal, about 20%, observed in both groups.

A possible explanation for these findings is that superior plates, regardless of their design, may cause discomfort due to their subcutaneous location and misfit to the bone, despite the advances in plate design [4,14,15]. Although it is not statistically significant, we found fewer females undergoing plate removal after clavicular fracture fixation in the SGP group compared to the FGP group, 14% and 21%, respectively. This is compatible with Jerry I Huang et al.’s conclusions, reporting that the pre-contoured anatomic clavicular plate appears to fit the S-shaped curvature on the superior surface of the majority of clavicles in male patients but may not be as conforming in white female patients. Here, we can assume that the new plate design of the SGP caused less irritability in this sub-group, and then we found a lower rate of plate removal [5].

While not statistically significant in our findings, lag screw use may be a potentially protective factor. The biomechanical rationale and prior studies suggest that lag screw application for stabilizing butterfly fragments or comminuted zones may add additional stability, possibly reducing micromotion and associated mechanical irritation. This theoretical advantage could potentially contribute to reduced hardware-related symptoms and, indirectly, a lower tendency for plate removal [16,17].

One recent study by Vancleef et al. [14] implemented a statistical shape model based on 120 clavicle 3D images. Their findings indicated that ten modes of variation were necessary to account for 95% of clavicular anatomy. Key variations included differences in clavicle length, sigmoid curvature, and medial morphology. The authors concluded that no off-the-shelf clavicle plate system could achieve a perfect fit for all patients [14].

In response to the anatomical variability of the clavicle bone, newer plate designs incorporate curvature adjustments based on patient height, offering three different plate sizes in the newer system (Figure 2) [8]. An intraoperative template is used on the reduced fracture to guide plate selection and achieve the best possible fit. However, authors observed that misfit still occurred in some cases, necessitating some compromises in plate placement. Given that only three sizes were available, and the selection is solely determined by patient height, this system’s improvement may be deemed insufficient to eliminate the need for implant removal. This is compatible with Vancleef et al.’s conclusions that, based on the identified variability in the clavicle’s anatomy, it seems unlikely that a clavicle plating system can fit the entire population.

Given the difficulties associated with achieving optimal plate fit for the superior clavicular surface, other fixation methods for midshaft clavicle fractures have been proposed. One option is positioning the plate on the anteroinferior surface of the clavicle. This approach theoretically reduces the likelihood of hardware protrusion due to the plate’s less subcutaneous location. However, the advantage of this technique in reducing hardware removal rates has not yet been demonstrated. To date, only one study has specifically addressed this issue, showing similar hardware removal rates for anteroinferior plates compared to superior plates [18,19,20]. In other studies, patient-reported implant prominence was nearly double in patients with a retained superior plate; however, implant removal occurred more frequently after superior plating but was not significant. The superior plating group showed a statistically shorter operative time. High rate of re-interventions with implant removal was documented in both groups [18,19,20].

An alternative to superior clavicular plating is the use of dual, orthogonally placed mini-fragment plates (2.4 mm and/or 2.7 mm). Biomechanically, these plates can provide stiffness comparable to that of a 3.5 mm plate, with similar clinical outcomes. The potential advantage of this approach is a lower reported implant removal rate. However, the drawbacks include higher implant costs and a longer surgical duration [21,22,23].

Another reported alternative to superior plate fixation is the use of intramedullary devices, such as flexible titanium nails. However, their application is generally limited to minimally displaced shaft fractures without comminution, such as AO/OTA B1 fractures. In our study, the majority of fractures (more than 50%) were classified as B2 and B3 [24]. While intramedullary fixation offers the advantage of smaller incisions, the risk of hardware migration can outweigh these benefits, making hardware removal a more complex procedure [25,26,27]. Studies have shown that patients undergoing plate fixation typically recover faster following surgery [28].

The analysis of reasons for hardware removal showed an interesting shift in the leading cause for implant removal. While first-generation 3.5 mm plates were primarily removed for traditional complications such as irritation (38.9%) and pain (25.0%), second-generation 2.7 mm plates showed a markedly different complication profile, with hardware prominence becoming the leading cause of removal (38.5% versus 8.3%, *p* = 0.024). Newer implant designs necessarily translate to improved patient outcomes and suggest that design modifications may inadvertently introduce novel complications while addressing traditional ones. The persistence of substantial removal rates (~20%) across both plate types, combined with this shift in complication patterns, indicates that current approaches to clavicle plate design may have reached a plateau in terms of clinical effectiveness [29,30]. Furthermore, the results support the need for more fundamental changes in fixation policies, such as the use of dual plates or alternative fixation methods, rather than continued refinement of single superior plate designs that appear to have inherent limitations regardless of manufacturing improvements [31,32].

The primary limitations of this study include its retrospective design and the relatively small sample size of patients treated with the newer plate fixation system. A possible explanation for the failure to demonstrate a significant advantage of the thinner, lower-profile plate is that, despite all procedures being performed by experienced fellowship-trained surgeons, there is still a learning curve associated with the application of these newer plating systems. Furthermore, a larger sample size is required to detect more subtle outcome differences.

Another potential bias in this study may stem from differing policies regarding implant removal across countries and cultures. Most of our patients were young and active, and a subcutaneous implant may interfere with vigorous physical activities. Additionally, surgeon policies regarding implant removal can vary significantly between regions. In some European countries, implant removal is routine [33], while in developing countries, this may not be the case. For example, one study notes that “routine removal of implants after fracture union constitutes a great waste of highly needed cash” [34].

In our experience, patients in our country are more likely to seek implant removal, although this observation requires further investigation.

## 5. Conclusions

Currently, despite it being a highly successful procedure with an over 98% union rate, the rates of superior clavicular plate removal remain significant, regardless of the implant design. Proper patient counseling before the index surgery is essential for managing expectations regarding potential hardware-related issues. Further studies exploring alternative treatment options may help reduce the rates of symptomatic hardware removal and improve patient outcomes.

## Figures and Tables

**Table 1:** Demographic data and information regarding the two groups. * Presence versus absence of lag screw/s. n = number.

	Group 1 (n = 184)	Group 2 (n = 67)	*p*-Value
**Age (SD)**	32.86 (13.64)	32.14 (13.15)	0.39
**Gender:**			0.75
**Female (n)**	16.85% (31)	14.92% (10)	
**Side of injury:**			0.47
**Left (n)**	51.63% (95)	47.76% (32)	
**Plate removal (n)**	20.1% (37)	20.9% (14)	0.86
**Mean days to removal (SD)**	331.5 (207.6)	341.02 (114.3)	0.83
**Type of fracture AO/OTA (n):**			0.86
**B1**	41.85% (77)	38.81% (26)	
**B2**	30.98% (57)	34.32% (23)	
**B3**	27.17% (50)	26.86% (18)	
Lag screw (SD) *	0.62 (0.79)	0.69 (0.75)	0.56

**Table 2:** Plate removal, comparison between the two groups. * Presence versus absence of lag screw/s. n = number.

	Group 1 (n = 37)	Group 2 (n = 14)	*p*-Value
**Age (SD)**	30.27 (12.51)	28.28 (11.73)	0.60
**Gender:**			0.56
**Female—(n)**	21.62% (8)	14.28% (2)	
**Side of injury:**			0.03
**Left—n**	10	14	
**Mean days to removal**	331.5	341.2	0.87
**Type of fracture AO:**			0.57
**B1**	18	6	
**B2**	11	3	
**B3**	8	5	
Lag screw (SD) *	0.40 (0.64)	0.57 (0.75)	0.47

**Table 3:** Hardware removal reasons. Other reasons include numbness, patient preference to remove metal implants, cosmetic concerns, and unspecified reasons.

Removal Reason	Total %	Group 1%	Group 2%	*p*-Value
Irritation	32.7	38.9	15.4	0.12
Pain	22.4	25	15.4	0.70
Hardware Prominence/Bulging	16.3	8.3	38.5	0.02
Nonunion	6.1	5.6	7.7	1
Infection	4.1	5.6	0	1
Limited Range of Motion	4.1	2.8	7.7	0.49
Other Reasons	14.3	13.9	15.4	1

## Data Availability

The raw data supporting the conclusions of this article will be made available by the authors on request.
